# An in vivo gene delivery approach for the isolation of reasonable numbers of type 2 innate lymphoid cells

**DOI:** 10.1016/j.mex.2020.101054

**Published:** 2020-09-10

**Authors:** Michael Frech, Lisa Knipfer, Stefan Wirtz, Mario M. Zaiss

**Affiliations:** a Department of Internal Medicine 3, Rheumatology and Immunology, Friedrich-Alexander-University Erlangen-Nürnberg (FAU) and Universitätsklinikum Erlangen, Erlangen, Germany; b Department of Internal Medicine 1, University of Erlangen-Nuremberg, Germany; c Deutsches Zentrum für Immuntherapie (DZI), Germany

**Keywords:** Group 2 innate lymphoid cells, Fluorescent activated cell sorting, Cell culture, Cytokines

## Abstract

Group 2 innate lymphoid cells (ILC2s) are a recently recognized subset of innate lymphocytes with crucial role in mucosal immunity and tissue homeostasis. Over the past decade, substantial advances in our understanding of ILC2 biology have established them as an essential element in innate and adaptive immunity. However, their relatively low abundance and laborious purification from mucosal tissues make their study difficult. Moreover, due to a lack of an ILC2-specific Cre mouse-line, adoptive transfer of ILC2s into ILC-deficient hosts is inevitable. Herein we describe an in-depth protocol for the induction, isolation, and expansion of murine ILC2s. By combining an in vivo gene delivery approach to boost ILC2 numbers and a cell culture strategy to expand isolated cells, large quantities of highly pure ILC2s can be obtained. The isolated cells maintain their phenotype and can be used for subsequent cell transfer or in vitro studies. In comparison to previous protocols, this approach is cost-effective and efficient with potential yield of more than 20 million ILC2s isolated per mouse.

• *Group 2 innate lymphoid cells (ILC2s) are extensively studied in mouse models and humans in recent years.*

• *Low abundance of ILC2s and current lack of specific ILC2 knockout mice makes in vivo research challenging.*

• *This method allows high and pure ILC2 numbers for in vitro or adoptive in vivo transfer experiments.*

## Graphical abstract

**Figure:**
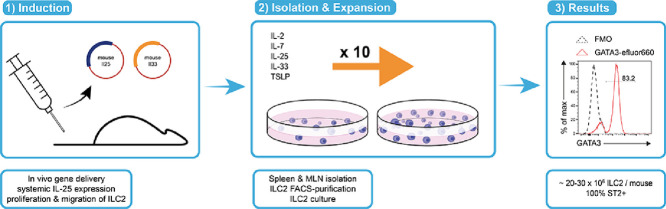

Specifications Table

**Table:** 

Subject Area:	Immunology and Microbiology
More specific subject area:	*Mucosal immunology*
Method name:	*Isolation and expansion of murine ILC2s*
Name and reference of original method:	[1] K. Moro, K.N. Ealey, H. Kabata, S. Koyasu, Isolation and analysis of group 2 innate lymphoid cells in mice, Nat Protoc. 10 (2015) 792–806. doi:10.1038/nprot.2015.047. [2]*C.U. Duerr, C.D.A. McCarthy, B.C. Mindt, M. Rubio, A.P. Meli, J. Pothlichet, M.M. Eva, J.-F. Gauchat, S.T. Qureshi, B.D. Mazer, K.L. Mossman, D. Malo, A.M. Gamero, S.M. Vidal, I.L. King, M. Sarfati, J.H. Fritz, Type I interferon restricts type 2 immunopathology through the regulation of group 2 innate lymphoid cells, Nat Immunol. 17 (2016) 65–75. doi:10.1038/ni.3308.*
Resource availability:	*If applicable, include links to resources necessary to reproduce the method (e.g. data, software, hardware, reagent)*

## Introduction

ILC2s are considered the innate counterpart of T helper 2 (Th2) cells, which is developmentally tied to high expression levels of Gata-binding protein 3 (GATA-3) [3]. They respond to epithelium-derived cytokines, such as TSLP, IL-25 and IL-33, and are involved in anti-parasite immunity, while in steady-state they are involved in tissue homeostasis in the small intestine, lungs and adipose tissue, in particular [4]. ILC2s are characterized by their ability to produce type 2-related cytokines such as IL-4, IL-5, IL-13 [5], [6], [7] as well as Amphiregulin (AREG) [8]. It was shown that the epithelium–derived cytokines IL-25 and IL-33 are crucial for the induction and activation of ILC2s [9], although it is increasingly appreciated that different populations of ILC2s are differentially responsive to these cytokines. Based on cytokine receptor expression and tissue tropism, two distinct groups of ILC2s were described. Natural ILC2s (nILC2s) that are present under homeostatic conditions are ST2+ and respond to IL-33, while inflammatory ILC2s (iILC2s), expressing high levels of IL17RB, preferentially arise upon IL-25 stimulation [10].

FACS-purification of defined immune cell populations is crucial for their investigation in immunological studies. However, reliable identification of ILC populations is dependent on lineage determining transcription factors, necessitating cell fixation. Moreover, isolation of ILC2s is limited by their low abundance. ILCs are vastly tissue-resident cells, deeply embedded into the fabric that further aggravates their purification. Cell isolation from non-lymphoid tissue requires laborious digestion that might lead to downregulated receptor expression and consequently hampers identification by flow cytometry. Here we provide a protocol that exploits the proliferation capacity of IL-25-responsive ILC2s upon systemic IL-25 challenge. We employ a hydrodynamic gene delivery (HGD) approach to cost-efficiently express high amounts of recombinant IL-25 and IL-33 *in vivo*. Owing to their rapid accumulation in mesenteric lymph nodes (mLN) and spleen upon systemic cytokine perception, ILC2s can then be easily sort-purified from these tissues, circumventing the need to process non-lymphoid tissues, such as the small intestinal lamina propria. Hence, about 3 million ILC2s per mouse can be routinely obtained using the provided gating strategy. Furthermore, following the culture condition provided herein, the number of ILC2s obtained per mouse can reach more than 20 million cells within two weeks. This method of ILC2 purification and expansion greatly facilitates the study of ILC2s by *e.g.* gene expression profiling, adoptive cell transfers and in vitro assays.

## Method Details

### Reagents

1. Digestion buffer: RPMI-1640 (Gibco), 2% FBS (Gibco), 0.25 mg/ml Collagenase B (Roche), 0.1 mg/ml DNaseI (Sigma Aldrich)
2. R10: RPMI-1640 (Gibco), 10% FBS (Gibco)
3. Staining buffer: PBS (Gibco), 2% FBS, 5 mM EDTA (Gibco)
4. ILC2 medium: DMEM (high glucose) (Gibco), 10% FBS, 1% penicillin/streptomycin (Merck), 1 mM sodium pyruvate (Gibco), 1 × non-essential amino acids (NEEA) (PAN Biotech), 50 µM 2-mercapto-Ethanol (Sigma Aldrich), 20 mM HEPES (pH 7.4) (Sigma Aldrich)
5. Ringer-solution (Ringer Fresenius, KABI France)
6. ACK buffer: 150 mM NH_4_Cl, 10 mM KHCO_3_, 0.1 mM Na_2_EDTA
7. IL-2 (Biolegend, cat. 575404), IL-33 (Immunotools, cat. 12340333), IL-25 (Immunotools, cat. 12340254), TSLP (Thermo Scientific, cat. 14-8798-80), IL-7 (Miltenyi, cat. 130-094-066)
8. ILC2 expansion medium: IL-2 (50 ng/ml) + IL-7 (50 ng/ml) + IL-25 (50 ng/ml) + IL-33 (50 ng/ml) + TSLP (20 ng/ml)
9. ILC2 resting medium: IL-2 (10 ng/ml) + IL-7 (10 ng/ml)

### Equipment

Flow Cytometer, Cytoflex (Beckman Coulter), Moflo Astrios EQ (Beckman Coulter)

Note: This list does not include any small generic laboratory equipment that is assumed to be available.

## Procedure

### Induction of ILC2s

The principle of in vivo transfection of naked DNA by hydrodynamic gene delivery (HGD) has been extensively described elsewhere [11]. Briefly, endotoxin-free plasmid DNA (pDNA) was applied by tail vein injection in one volume of Ringer-solution corresponding to 10% of the individual mouse body weight. We use 6 µg of plasmid encoding mouse IL-25 [12,13].

### Isolation

*Note: Since cells are to be put in culture, perform all steps using aseptic techniques.*

1. Prepare a 24-well plate with ice-cold PBS before beginning the experiment and place on ice.
2. Per mouse, prepare one Eppendorf tube containing 1 ml digestion buffer and pre-warm to 37°C.
3. Euthanize the mouse by CO_2_ inhalation or cervical dislocation.
4. *Optional:* draw blood for later cytokine detection by ELISA.
5. Harvest the mesenteric lymph nodes and the spleen into the ice-cold PBS and chill on ice (see Note 1).
6. Cut organs into small pieces and transfer into pre-warmed digestion buffer.
7. Incubate the samples at 37°C for 15 min.
8. Place a 70 µm cell strainer onto a 50 ml conical tube and pre-wet with 5 ml ice-cold staining buffer.
9. Transfer the samples onto the cell strainer and cut into smaller pieces (*see* Note 2).
10. Mash the tissue through the cell strainer using the plunger of a 3 ml syringe.
11. Rinse the cell strainer with 10 ml staining buffer.
12. Top up to 50 ml with staining buffer and centrifuge for 10 min at 400 × g, 4°C
13. Discard the supernatant and resuspend in 1 ml ACK buffer (see Note 3).
14. Add another 4 ml ACK buffer and incubate for 10 min at room temperature (RT) (see Note 4).
15. Top up with ice-cold staining buffer and centrifuge for 10 min at 400 × g, 4°C.
16. Decant the supernatant. Resuspend in 5 ml staining buffer and filter cell suspension through 70 µm cell strainer. Rinse the tube with additional 5 ml of staining buffer and pass through the same strainer.
17. Count the cells using the method of your choice.
18. Centrifuge the samples for 10 min at 400 × g, 4°C. Decant the supernatant.
19. Adjust the cell concentration to 2 × 10^8^ cells/ml by resuspending in the appropriate volume of staining buffer containing fc block. Incubate on ice for 10 min.
20. During that time, prepare a 2 × antibody mix according to Table 1 (Note 5):

**Table 1:** 

Antibody	Conjugate	Company	clone	dilution factor for 2x mix
CD3	FITC	Biolegend	17A2	300
B220	FITC	Miltenyi Biotec	REA755	50
CD5	FITC	Miltenyi Biotec	REA421	20
NK1.1	PE-Vio770	Miltenyi Biotec	PK136	50
CD11b	APC-Vio770	Miltenyi Biotec	REA592	20
CD11c	APC-Vio770	Miltenyi Biotec	N418	50
KLRG1	PE	Miltenyi Biotec	2F1	50
ICOS	VioBlue	Miltenyi Biotec	7E.17G9	20
FceR1a	PE-Cy7	Invitrogen	MAR-1	300
21. Add 1 volume to each sample and incubate for 20 min at 4°C in the dark.
22. Top up to 50 ml with staining buffer and centrifuge for 10 min at 400 ×  g, 4°C. Decant supernatant.
23. Resuspend the cell pellet with 1 ml staining buffer and pass through 30 µm cell strainer into a 15 ml conical tube. Rinse the tube with another 5 ml and pass through the same strainer. Top up with staining buffer and centrifuge for 5 min at 400 × g, 4°C.
24. Resuspend the cell pellet in 2 ml staining buffer and proceed to cell sorting. For each sample provide the FACS-operator with one 15 ml collection tube with 2 ml of ILC2 resting medium.

*Culture*

1. Pre-warm ILC2 expansion medium.
2. After cell-sorting and purity check, centrifuge the samples for 10 min at 400 × g, 4°C.
3. Seed 0.5 × 10^6^ cells per well in a 24-well plate and culture with 1 ml ILC2 expansion medium.
4. Replenish the medium every 36–48 h and passage the cells as they achieve higher density.
5. Prior to downstream experiments, replace medium with ILC2 resting medium for two days (see Note 6).

*Notes*

1. If the hydrodynamic injection was successful, enlarged mLNs and splenomegaly can be observed. Moreover, the gastrointestinal tract typically exhibits a bloated/well hydrated phenotype. Generally, cell suspensions of boosted mice are more “sticky” compared to naïve mice. Hence, in case necrosis is observed, these parts should be thoroughly removed. In our experience, the occurrence of necrosis results in drastically increased clumping of the cells, which might hinder stable cell sorting. Therefore, we recommend injecting at least one backup mouse per condition. In many cases, however, repeated filtering can help.
2. Thorough cutting of the samples will result in significantly increased cell yield.
3. We prefer to resuspend in 1 ml using a 1 ml pipette tip, and then add another 4 ml of buffer, since careful resuspension is necessary for efficient red blood cell lysis.
4. Pipetting up and down half-way through the incubation can help avoid cell clumping.
5. In order to save parameters, antibodies specific for lineage markers can be combined within one dump channel. However, we strongly advice to separately dump myeloid and lymphoid cells, as this facilitates troubleshooting in case of poor staining index. Also note that in some mouse strains, such as Balb/c, NK cells do not express NK1.1; CD49b can be used instead.
6. This step will put the ILC2s back from an “overactivated” into a quiescent, responsive state.

## Validation

**Fig. 1:**
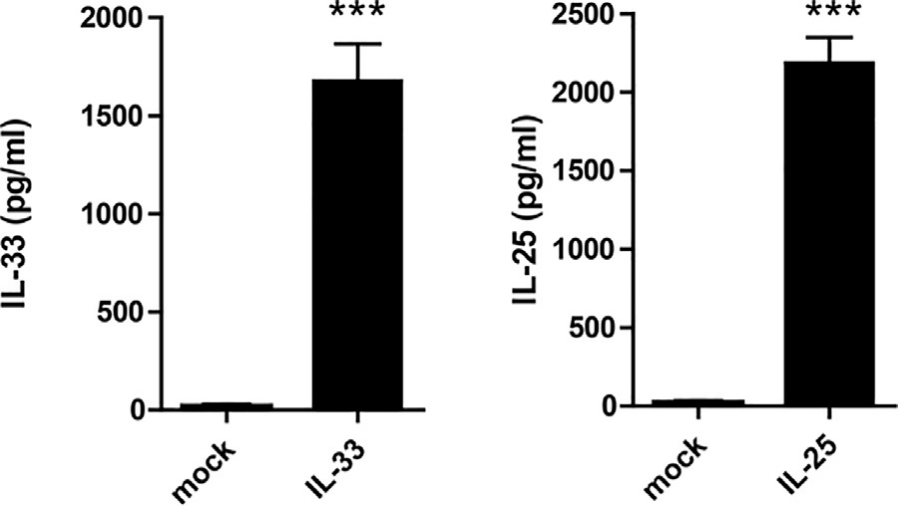
Serum IL-33 and IL-25 expression after HGD injection of 4µg IL-25 and IL-33 3 dpi. Data are expressed as mean ± SD; ***p < 0.001

**Fig. 2:**
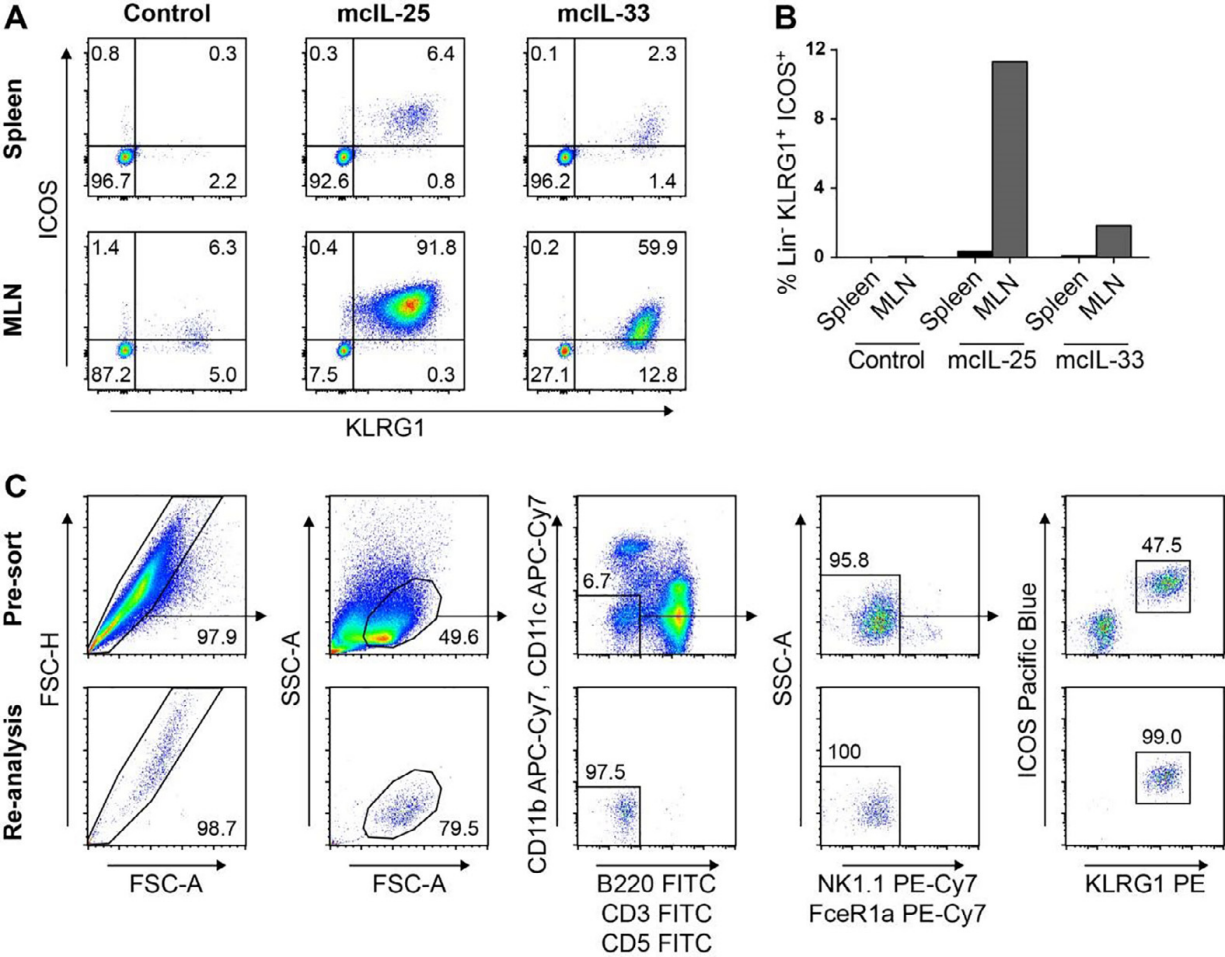
Analysis of ILC2s by flow cytometry after HGD with mcIL-25 and mcIL-33. (A) Flow cytometric analysis of KLRG1+ICOS+ ILC2s from spleen and mLN of animals 3 dpi with mcIL-25 or mcIL-33 and control. Gated on Lineage negative cells. (B) Frequencies of Lineage negative KLRG1+ICOS+ cells of control, mcIL-25 or mcIL-33 injected mice from spleen and mLN 3 dpi. (C) Gating strategy for the purification of ILC2s 3 days post HGD with mcIL-25 and re-analysis of the obtained cells after purification.

A successful HGD injection with mcIL-33 and mcIL-25 results in significantly increased serum concentrations of IL-33 and IL-25 (Fig. 1). To interrogate their efficiencies in recruiting ILC2s cells to spleen and mLN, we performed FACS analysis three days post injection (dpi). We found substantially increased ILC2 numbers in both spleen and mLN after injection of either plasmid, as determined by lin-ICOS+KLRG1+ cells (Fig. 2A and B). In spite of the much higher increase of ILC2 frequency in mLN compared to spleen, it is advantageous to sort from both organs owing to the higher splenic cellularity. Furthermore, mcIL-25 injection results in a higher accumulation of ILC2s than mcIL-33 in both spleen and mLN (Fig. 2A and B). Therefore, we recommend use mcIL-25 or a combination of mcIL-25 and mcIL-33 for the induction of ILC2s. Of note, we also assessed ILC2 frequencies at later time points (data not shown), however, due to an accumulation of other immune cells (e.g. eosinophils), 3 dpi were found optimal.

After successful vector delivery and cell isolation, one can sort-purify - on average - three million ILC2s per mouse. A gating strategy is provided in Fig. 2C. First, doublets should be excluded, followed by gating on lymphocytes. Within the lymphocytes gate, myeloid and lymphoid cells can be excluded using the indicated parameters. Of those double negative cells, NK cells should be eliminated, as they exhibit a high degree of autofluorescence, leading to a potential false positive signal. Finally, ILC2s can be identified by their strong ICOS and KLRG1 expression. Prior to in vitro expansion, the described target population is between 80% and 90% GATA3+ (Fig. S1B).

**Fig. 3:**
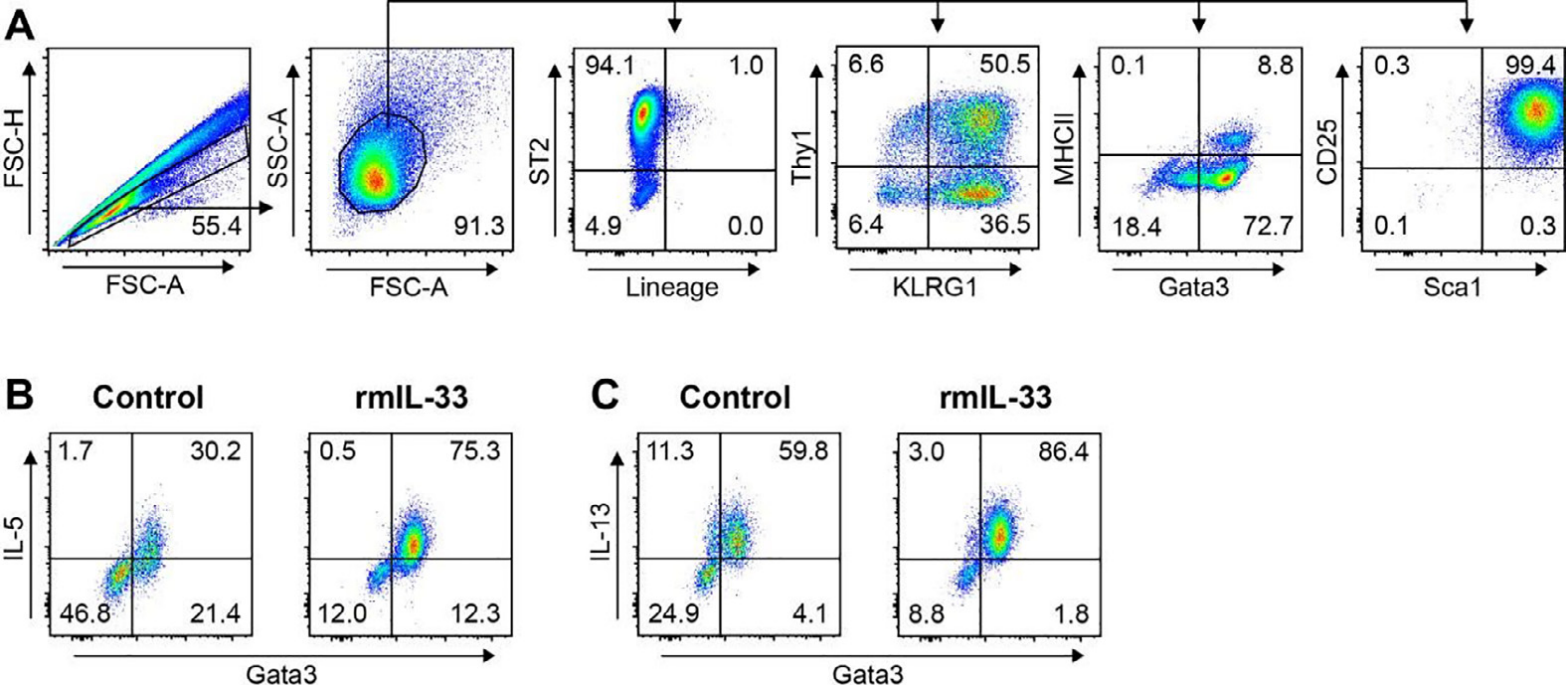
Phenotypic characterization of ILC2s after *in vitro* expansion. (A) ILC2s were *in vitro* expanded and stained for the indicated markers. (B-C) IL-33 stimulated and unstimulated (control) ILC2s were stained for intracellular IL-5 (B) and IL-13 (C) production.

After 14 days in culture as described above, the number of ILC2s reaches more than 20 million cells per mouse. Of these, we usually observe at least 80% and up to 99% GATA3+ (data not shown). To further characterize the phenotype of ILC2s after culture, we analyzed surface marker expression by flow cytometry. It was reported that iILC2s are rare in naïve mice but arise quickly upon IL-25 stimulation [10]. These ILC2s have high levels of KLRG1 but lack ST2. In stark contrast to nILC2s, iILC2s show high expansion in number both *in vitro* and *in vivo*. In fact, iILC2s were shown to give rise to nILC2s during helminth infection [10]. Thus, iILC2s are designated an important source for ILC2s during infections. Indeed, the frequency of *ex-vivo* ILC2s expressing the IL-33 receptor gamma chain ST2 after mcIL-25 injection is only about 5% (Fig. S1), but is highly upregulated *in vitro* (Fig. 3A), supporting the idea of a transient nILC2 progenitor. Since our cytokine cocktail for *in vitro* expansion also contains the alarmin IL-33, we obtain a mixed population of iILC2s and nILC2s that also expresses high levels of IL17RB (data not shown). Moreover, *in vitro* expanded ILC2s were KLRG1 high and also stained double positive for Sca1 and CD25. Closer phenotyping revealed that about 50% of ILC2s were Thy1+. Recently, ILC2s were shown to express MHCII [14]. We observed about 80% GATA-3+ ILC2s, of which 10% were MHCII positive. Interestingly, the frequency of MHCII+ ILC2s might be higher *in vivo*, since this was partially attributed to trogocytosis [14]. We further analyzed their cytokine expression after stimulation with IL-33. In accordance with other reports, ILC2s produced large amounts of the type 2 cytokines IL-5 (Fig. 3B) and IL-13 (Fig. 3C) after IL-33 stimulation [9].

## Declaration of Competing Interest

The authors declare no conflict of interest.

The authors declare that they have no known competing financial interests or personal relationships that could have appeared to influence the work reported in this paper.

## Appendix Supplementary materials

Image, application 1
