# Supplementary material for: An in vivo gene delivery approach for the isolation of reasonable numbers of type 2 innate lymphoid cells
Source: MethodsX. 2020 Sep 10;7:101054. doi: 10.1016/j.mex.2020.101054 (PMC7509459; doi:10.1016/j.mex.2020.101054)
Supplement: Supplementary file 1 [file mmc1.docx]

*
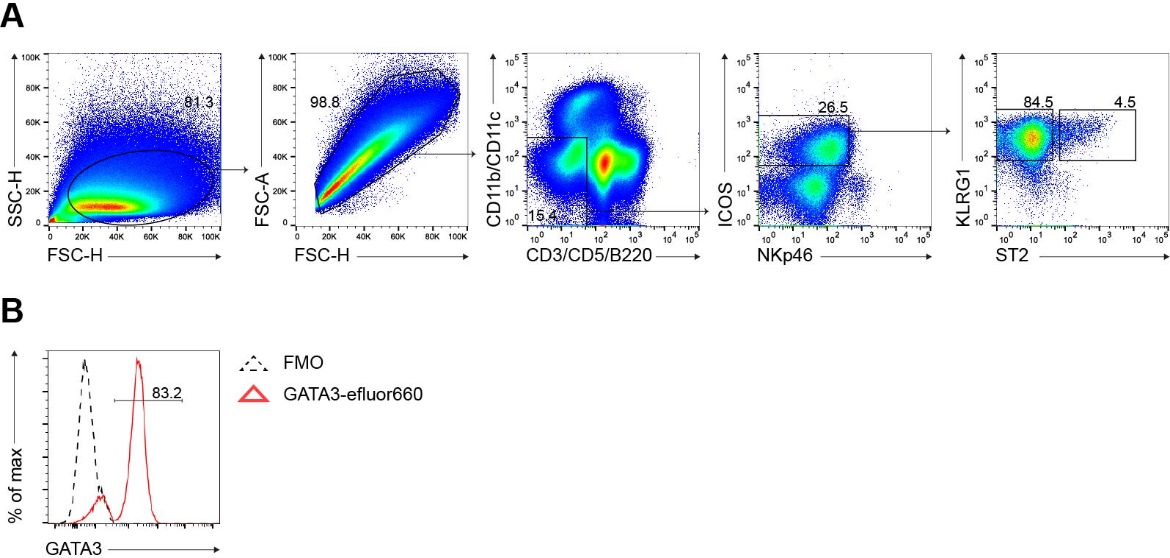
*

**Supplemental Figure 1:** Analysis of ILC2s by flow cytometry after HGD with mcIL-25 and mcIL-33. **(A)** Flow cytometric analysis of ILRG1+ST2+/- ILC2s from spleen and mLN of animals 3 dpi with mcIL-25 HGD. **(B)** Sorted KLRG1+ST2+/- ILC2s were stained for the expression of GATA3
